# Acceptability and Feasibility of a Self-Directed Health Priorities Program for Southern Older African Americans

**DOI:** 10.1093/geroni/igaf122.2023

**Published:** 2025-12-31

**Authors:** Deborah Ejem, Marie Bakitas, Raegan Durant, Tamara Nix Parker, Kwaku Duah Oppong, J Odom, Kenneth Boockvar, Mary Tinetti

**Affiliations:** The University of Alabama at Birmingham, Birmingham, Alabama, United States; The University of Alabama at Birmingham, Birmingham, Alabama, United States; The University of Alabama at Birmingham Heersink School of Medicine, Birmingham, Alabama, United States; The University of Alabama at Birmingham School of Nursing, Birimingham, Alabama, United States; The University of Texas at Austin, Austin, Texas, United States; The University of Alabama at Birmingham School of Nursing, Birmingham, Alaska, United States; The University of Alabama at Birmingham, Birmingham, Alabama, United States; Yale University, New Haven, Connecticut, United States

## Abstract

Acceptability and feasibility of a self-directed health priorities program for Southern older African Americans were assessed in a mixed-methods formative evaluation (NIH Stage 1a). The study explored patient and family caregiver (FCG) experiences with the “My Health Priorities” (MHP) web-based program, a component of the Patient Priorities Care Approach. Fifteen older African Americans (≥65 years, ≥2 chronic conditions) and their adult African American FCGs (≥18 years) from a southeastern U.S. primary care clinic participated. Thematic analysis of interviews and usability assessments via the System Usability Scale (SUS) were conducted. Patients generally found the program acceptable but faced navigation challenges on non-computer devices. Recommendations included culturally adapting avatars, optimizing mobile functionality, and providing strategies for patient-clinician interactions. Patients rated usability at 75.3 ± 14.6 (good), whereas FCGs rated it at 30.1 ± 4.3 (poor). Study measures took 30 minutes to complete, while the intervention took 60 minutes, with 81% of study measures completed. Findings suggest that web-based programs may be acceptable for African American patients and caregivers, but usability and cultural relevance require improvement. Differences in patient and caregiver usability ratings highlight the need for tailored design considerations.

